# A lower starting point for the medial cut increases the posterior slope in opening-wedge high tibial osteotomy: a cadaveric study

**DOI:** 10.1186/s40634-022-00562-z

**Published:** 2022-12-29

**Authors:** Juan Ignacio Erquicia, Sergi Gil-Gonzalez, Maximiliano Ibañez, Joan Leal-Blanquet, Andrés Combalia, Juan Carlos Monllau, Xavier Pelfort

**Affiliations:** 1 Department of Orthopedic Surgery and Traumatology, Althaia Xarxa Assistencial Universitària de Manresa, Carrer Dr. Joan Soler, 1-3, 08243 Manresa, Spain; 2 IMOVE, Mi Tres Torres, Av. Via Augusta, 281, 08017 Barcelona, Spain; 3 Department of Orthopedic Surgery and Traumatology, Consorci Corporació Sanitària Parc Taulí. Universitat Autònoma de Barcelona (UAB), Parc del Taulí, 1, 08208 Sabadell, Spain; 4 ICATME. Hospital Universitari Dexeus, Universitat Autònoma de Barcelona (UAB), Carrer de Sabino Arana 5, 08028 Barcelona, Spain; 5 Departament de Cirurgia i Especialitats Medicoquirúrgiques, Facultat de Medicina i Ciències de la Salut, Universitat de Barcelona (UB), Carrer Casanova 143, 08036 Barcelona, Spain; 6 Facultat de Medicina i Ciències de la Salut, Universitat de Barcelona (UB), Carrer Casanova 143, 08036 Barcelona, Spain; 7 Department of Orthopedic Surgery and Traumatology. Hospital del Mar, Universitat Autònoma de Barcelona (UAB), Passeig Marítim, 25, 08003 Barcelona, Spain

**Keywords:** Anterior cruciate ligament, Cadaveric study, Lateral knee X-ray, Medial starting point, Posterior tibial slope, Opening-wedge high tibial osteotomy, Osteotomy, Proximal anatomical axis

## Abstract

**Purpose:**

The objective of this study was to evaluate the effects on the posterior tibial slope of different distances from the joint line to start the osteotomy and of varying the placement of the opening wedge in high tibial osteotomy.

Starting the osteotomy more distally and an incorrect location for the tibial opening wedge were hypothesized to increase the posterior tibial slope.

**Methods:**

A cadaveric study was conducted using 12 knees divided into two groups based on the distance from the joint line to the start of the osteotomy: 3 and 4 cm. The preintervention posterior tibial slope was measured radiologically. Once the osteotomy was performed, the medial cortex of the tibia was divided into anteromedial, medial, and posteromedial thirds. A 10° opening wedge was sequentially placed in each third, and the effect on the posterior tibial slope was evaluated radiographically.

Results: Significant changes were observed only in the 3-cm group (*p* = 0.02) when the wedge was placed in the anteromedial zone. In contrast, in the 4-cm group, significant differences were observed when the opening wedge was placed at both the medial (*p* = 0.04) and anteromedial (*p* = 0.012) zones.

**Conclusion:**

Correct control of the posterior tibial slope can be achieved by avoiding a low point when beginning the osteotomy and placing the opening wedge in the posteromedial third of the tibia when performing an opening-wedge high tibial osteotomy.

**Level of evidence:**

Controlled laboratory study.

## Background

Proximal high tibial osteotomy (HTO) is the most widely accepted treatment for physically active patients with initial stages of medial femorotibial compartment degeneration and varus alignment of the limb [1]. Due to its particular advantages, opening-wedge high tibial osteotomy (OWHTO) has become more widely used in recent years than lateral closing-wedge osteotomies. Among these advantages, the lack of a need to perform an osteotomy of the fibular head with the subsequent risk of neurovascular injury [2] and less surgical exposure without muscle detachment have been cited [3]. In addition, the effect of OWHTO on the tibial metaphysis comparatively facilitates the performance of future knee arthroplasty [4].

However, OWHTO is not without potential disadvantages, including an increase in the posterior tibial slope (PTS) and a decrease in the patellar height (PH) [5].

Recently, the effects of the PTS on the biomechanics and stability of the knee [6, 7] have been described, as well as the tension exerted on both native and reconstructed cruciate ligaments [8–10]. Clinical and biomechanical studies have reported that the tension on the native or reconstructed anterior cruciate ligament (ACL) increases significantly after minimal changes in the PTS of only 2°, increasing the risk of failure [11, 12]. It is believed that the triangular cross-section of the tibia at this level as well as the way the osteotomy gap is produced highly influence the increase in the PTS [13, 14]. However, the inclination of the osteotomy plane has not been investigated.

The objective of this study was to evaluate the effect on the PTS of modifying the distance from the joint line where the osteotomy begins in the medial cortex of the tibia and of varying the placement of the opening wedge from posterior to anterior.

An increase in the PTS was hypothesized to be observed both when distalizing the point where the osteotomy begins and when anteriorizing the tibial opening wedge.

### Materials and methods

For this analysis, 12 cadaveric human knees were used, stored at a temperature of − 18 °C, and thawed at room temperature for 24 to 36 hours prior to the procedure, during which they were wrapped in gauze moistened with saline solution. Each knee included the distal two-thirds of the femur and the proximal two-thirds of the tibia and fibula. The knees were macroscopically evaluated and showed no signs of having undergone surgery. In addition, prior to use, the absence of local radiographical bone lesions or any significant bone malformation that might affect the anatomy was confirmed.

The knees were randomly divided into two groups based on the distance from the start of the osteotomy to the joint line: 3 and 4 cm (cm). All surgical procedures were performed by the same team of two senior orthopaedic surgeons.

The study was approved by the clinical research ethics committee of our institution (protocol number PR260/21-CSA PR22/2021).

#### Radiographic technique

Prior to performing the osteotomy, the radiographic procedure was standardized to achieve greater reproducibility of the technique and to ensure that no magnification errors would influence the measurements [15]. Initially, each knee was placed extended in the anteroposterior (AP) plane, and the image was focused on the centre of the tibial shaft. One-third of the fibular head was covered by the lateral tibial plateau to ensure a correct AP view. This AP view was used to measure the width (mediolateral) of the tibial epiphysis by creating a parallel line that passed 5 mm distal to the joint space. In addition, the mediolateral size of the tibia was measured 3 and 4 cm from the joint line using a line parallel to that described above.

Subsequently, a strict lateral knee X-ray, where both femoral condyle contours perfectly overlapped, was performed at 30° of flexion. This projection was used to measure the PTS.

#### Radiological PTS evaluation

**Fig. 1:**
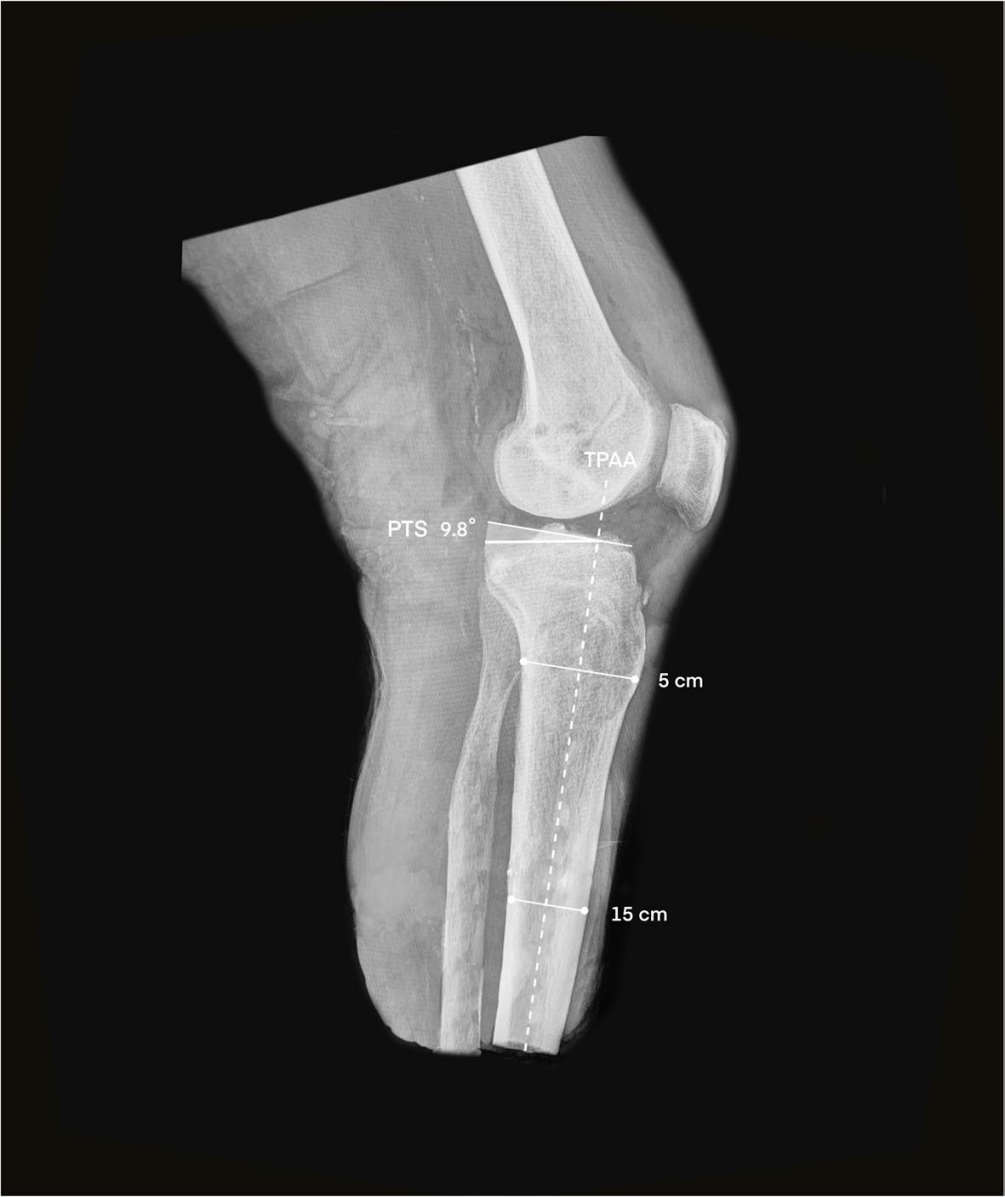
Tibial slope measurement on lateral radiographs. The tibial proximal anatomic axis (TPAA) is drawn connecting the midpoints between the anterior and posterior tibial cortex at five and 15 cm distances to the joint line. A reference line is drawn perpendicular to this at the level of the tibiofemoral joint. The mean between the inclinations of both plateaus is also shown. The angle of this line to the reference line is defined as the posterior tibial slope (PTS)

The medial and lateral tibial plateaus were initially identified to establish the PTS. The mean between the inclinations of both plateaus was used as previously published [16]. In measuring both tibial plateaus, the existence of osteophytes was excluded. In the same lateral view of the knee, the longitudinal component of the PTS was established by following the proximal anatomical axis of the tibia. For this analysis, two points were identified at 5 and 15 cm distal to the joint line. The midpoint of these levels, namely, the distance between the anterior and posterior cortex of the tibia, was identified. These midpoints were connected with a line that determined the longitudinal axis mentioned above and are shown in Fig. 1 [17, 18]. The same protocol was used for the radiographic technique and measurement of the PTS after osteotomy.

All radiographic measurements were performed by two orthopaedic surgeons using the picture archiving and communications system (PACS) (Centricity Enterprise Web V3.0; GE Healthcare).

### Osteotomy technique

**Fig. 2:**
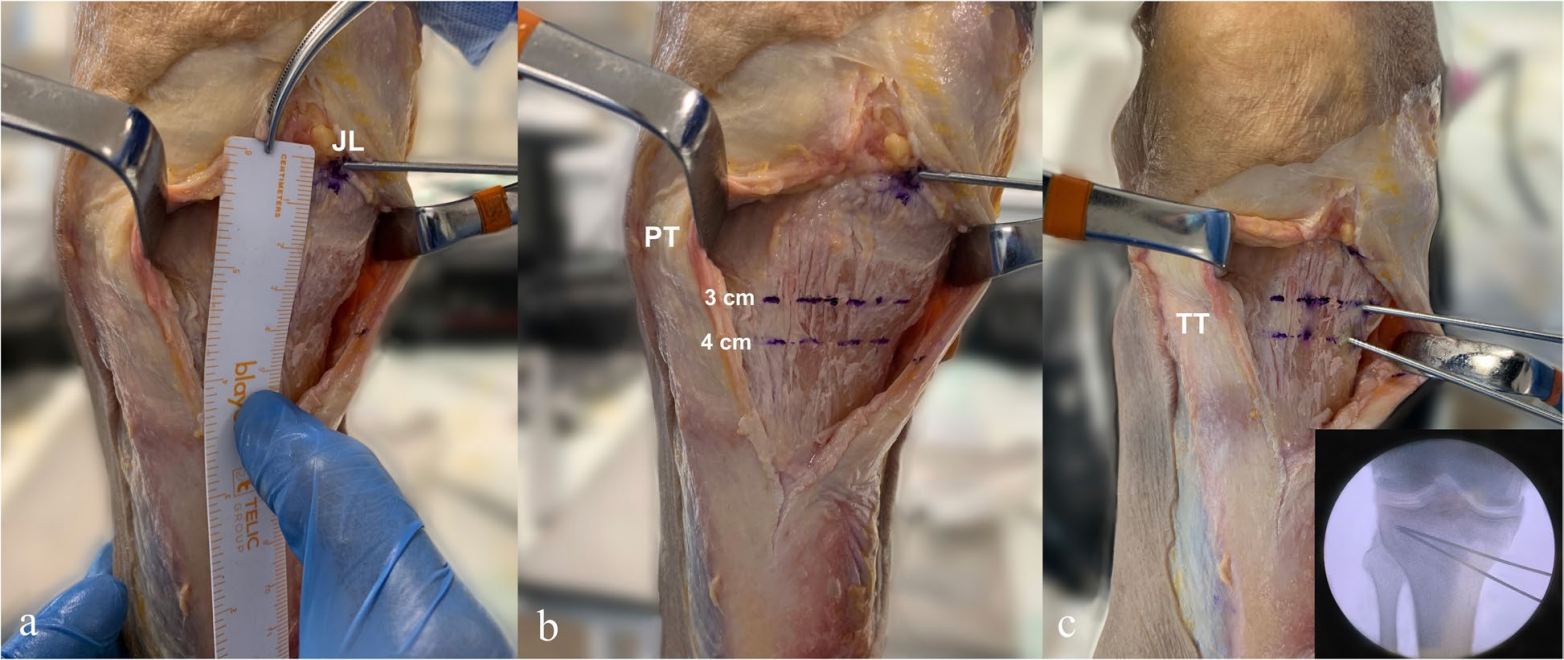
Medial starting point. Anteromedial view of the right knee. (**a**) Kirschner wire located at the level of the joint line and measurement of the site where the osteotomy will begin. JL, joint line. (**b**) The methodology is graphed according the sites where the osteotomy will begin according to randomization. PT, patellar tendon (**c**) Two 2.4-mm wires are introduced at 3 and 4 cm, which will serve as a guide for performing the osteotomy for the 3 and 4 cm groups, respectively. A wide dissection of skin and subcutaneous tissue is performed to facilitate understanding of the image. TT, tibial tuberosity

A 6- to 8-cm longitudinal skin incision was made in the anteromedial and proximal area of the leg midway between the anterior tuberosity and the posteromedial edge of the tibia. After dissecting the subcutaneous tissue, the tendons of the pes anserinus were incised longitudinally in a single plane at the level of the anterior margin of the medial collateral ligament. Both structures were carefully retracted posteriorly with the help of a Hohman retractor. The patellar tendon was identified and protected with a retractor. After the metaphyseal-diaphyseal transition zone of the tibia was identified, according to the randomization group (namely, 3 or 4 cm) (Fig. 2), a 2.4-mm Kirschner wire was introduced under fluoroscopic control (C-ARM Fluoroscopy 4400, FM Control, Alava, Spain) in the medial cortex aiming posterolaterally towards the proximal end of the fibular head. The tip of the wire was located 1.5 cm distal to the joint space and 1 cm medial to the lateral cortex. Following wire insertion, the osteotomy was performed, and special attention was paid to maintaining perpendicularity with respect to the sagittal long axis of the bone. Initially, the procedure was started with a saw and continued with a calibrated osteotome, with particular focus on completion of the osteotomy in the posterolateral region of the tibia and preserving one cm of bone in the lateral tibial cortex, which acts as a hinge [14, 19]. Subsequently, a lamina spreader was used to verify a sufficient opening to progressively place a 10° opening wedge (Newclip Technics, Haute-Goulaine, France).

**Fig. 3:**
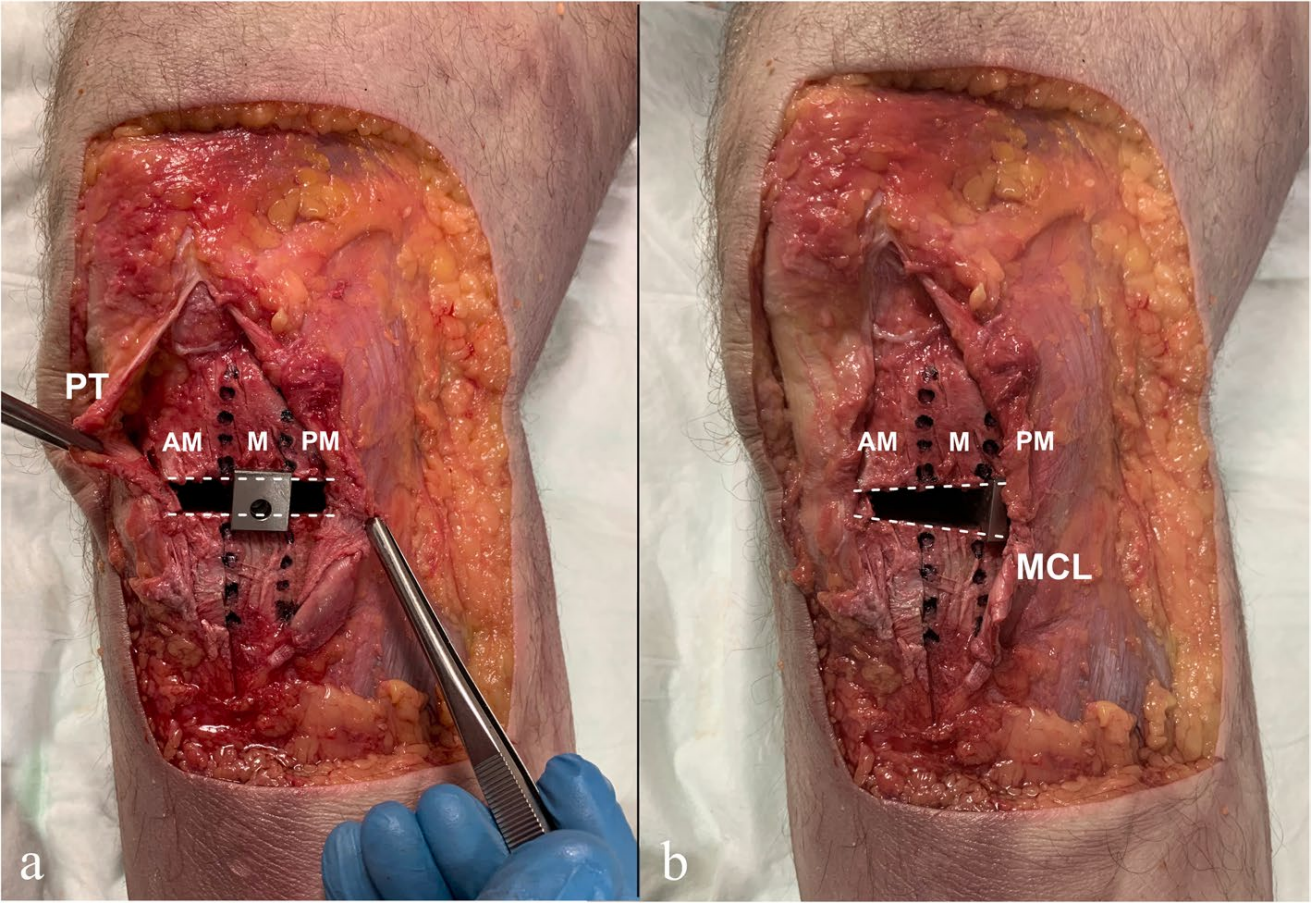
Anteromedial view of the right knee, 4 cm case. The placement of the opening wedge in the M third of the tibia generates a parallel gap (**a**). In contrast, when the wedge is located in the PM third of the tibia, the result is an asymmetric gap. The extent to which the medial collateral ligament (MCL) may hinder the posterior placement of the opening wedge is also shown (**b**). PT, patellar tendon; AM, anteromedial; M, medial; PM, posteromedial

**Fig. 4:**
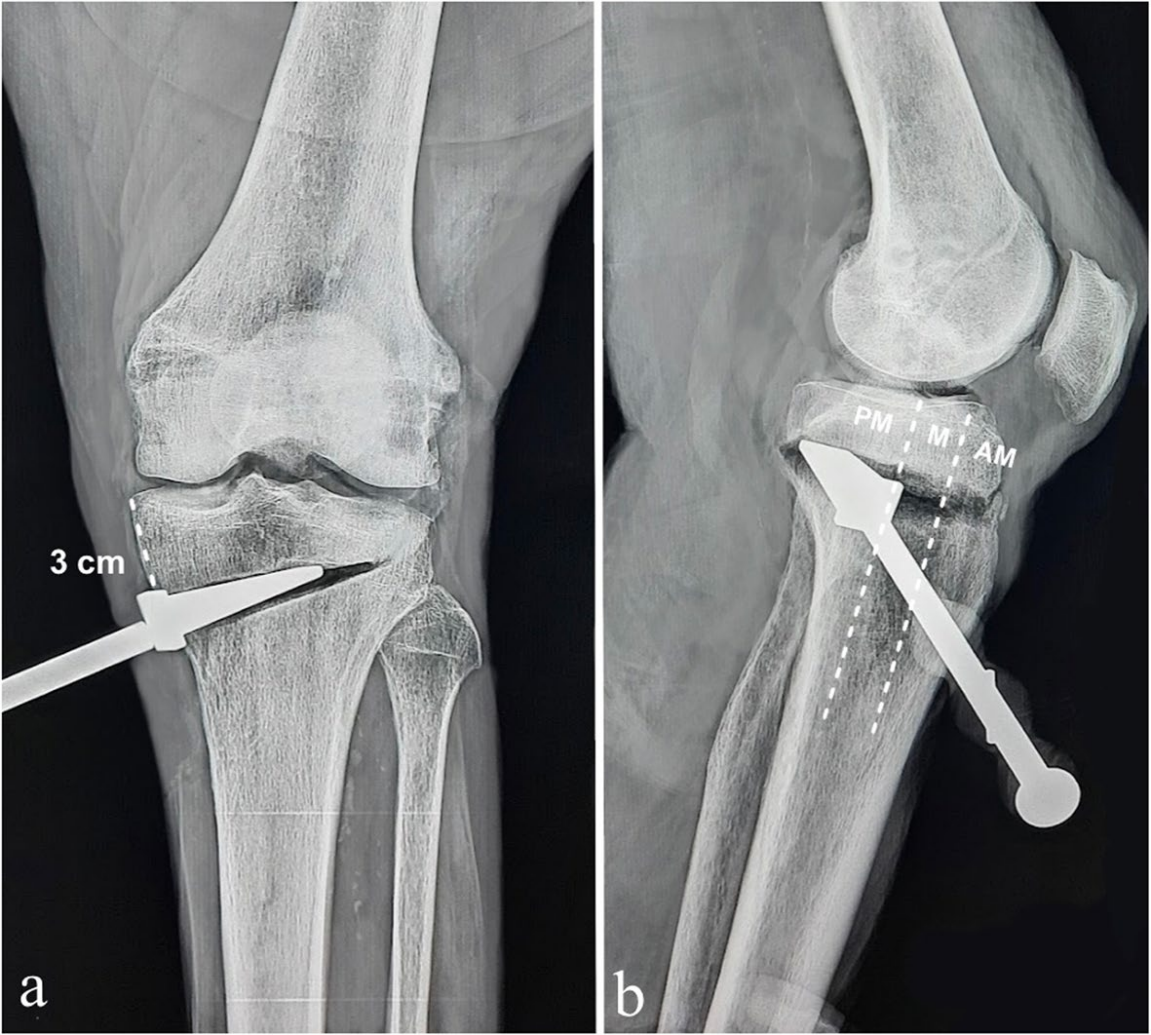
Radiographic control in a left knee, 3 cm case. Anteroposterior view (**a**) and lateral knee projection (**b**) with an opening wedge in the PM third location. AM, anteromedial; M, medial; PM, posteromedial

After the anteromedial, medial and posteromedial (AM, M and PM, respectively) thirds of the medial cortex of the tibia were identified, the same 10° opening wedge was placed in each third (Fig. 3), and radiographic projections were taken as explained previously (Fig. 4).

Internal fixation was not performed with plates since the objective of the study was to evaluate the possible variations in the tibial slope and not the stability provided by an implant.

### Statistical analysis

A descriptive analysis of the study variables was performed. Radiographic parameters were measured twice by two independent orthopaedic surgeons at 6-week intervals. The inter- and intrarater reliability for the radiographic measurements was assessed by calculating the intraclass correlation coefficients (ICCs). The ICC values were interpreted as follows: ICC < 0.40, poor agreement; 0.4 > ICC < 0.75, fair to good agreement; and ICC > 0.75, excellent agreement [20, 21].

The chi-square or Fisher’s exact test was used to compare categorical variables, and the t test was used for continuous variables. Pearson’s correlation coefficient was calculated to compare the PTS measurements at the different sites where the opening was performed and the starting point of the osteotomy.

The sample size was calculated a priori. With six subjects per group (*n* = 12), a statistical power of 80% was obtained to detect a difference ≥ 2° between the different tests [22], accepting an alpha risk of 0.05 and a beta risk of 0.2 in a bilateral comparison.

Because this is a cadaveric study, a loss to follow-up rate of 0% was estimated. All data were analysed with SPSS Statistics (v 21; IBM), with statistical significance was established for *p* < 0.05.

## Results

Eight of the knees were obtained from women and four from men. Six were right knees, and six were left knees. The mean age was 65.3 ± 13.3 years. The overall descriptive analysis is presented in Table 1.

**Table 1:** Descriptive statistics

	N	Minimum	Maximum	Mean	SD
**ML**	12	77.6 mm	97.8 mm	88.6 mm	5.3 mm
**ML 3 cm**	12	68.8 mm	84.9 mm	77.3 mm	3.3 mm
**ML 4 cm**	12	54.3 mm	65.8 mm	59.6 mm	5.1 mm
**PTS preop.**	12	4.3°	12.1°	7°	2.3°
**PTS PM**	12	2.9°	12.5°	7.2°	2.4°
**PTS M**	12	4.4°	14.2°	8.6°	2.8°
**PTS AM**	12	6.9°	17.5°	11.2°	3.3°

ML, mediolateral; ML 3 cm, mediolateral 3 cm from the joint line; ML 4 cm, mediolateral 4 cm from the joint line; SD, standard deviation; PTS, posterior tibial slope; PM, posteromedial; M, medial; AM, anteromedial

### Global measurement of the PTS (n = 12)

**Fig. 5:**
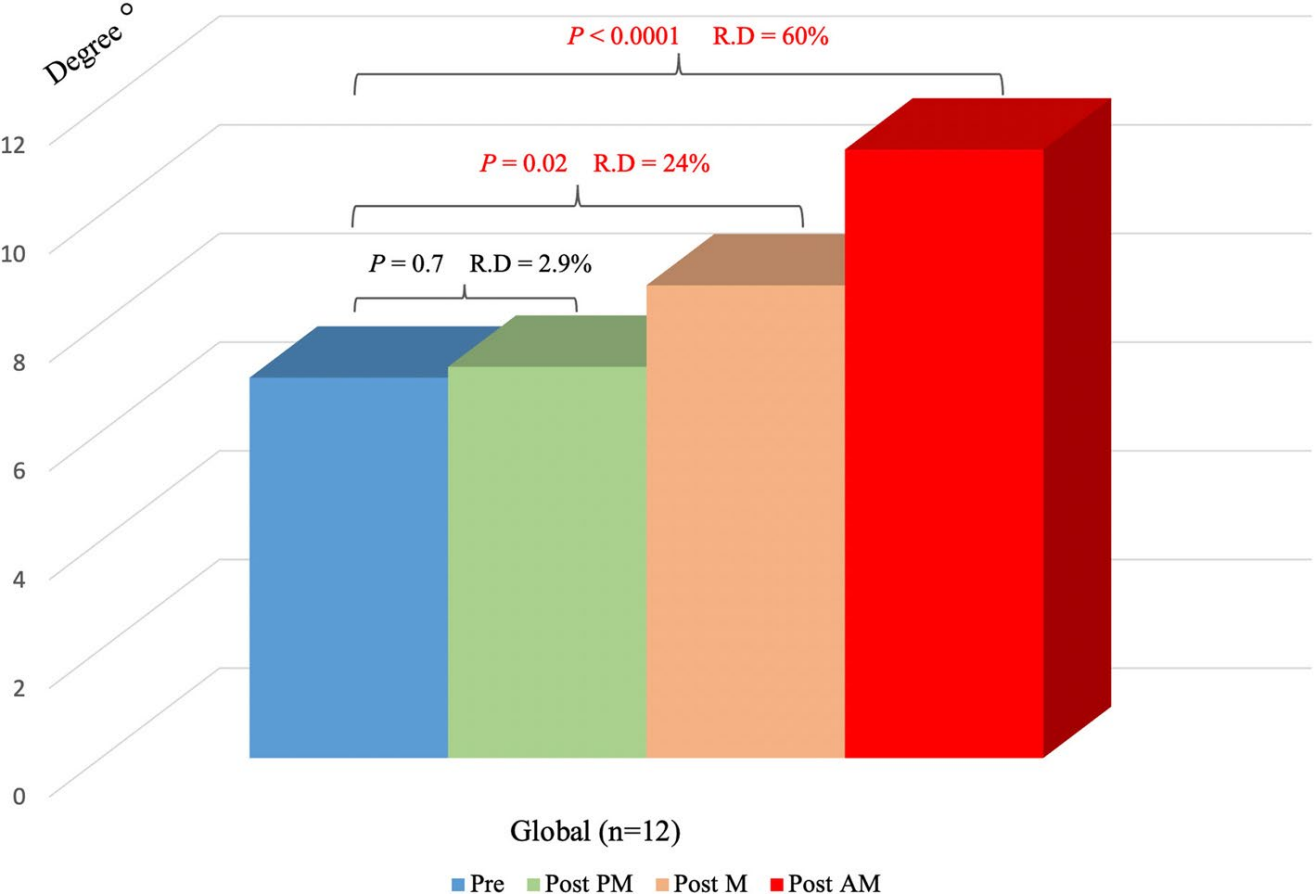
Preoperative vs postoperative PTS (global 3 & 4 cm, *n* = 12). The red text indicates significant changes. R. D, relative difference; AM, anteromedial; M, medial; PM, posteromedial

The mean preoperative PTS was not significantly different from the postoperative value when placing the opening wedge at the PM level (preoperative 7.0 ± 2.3° and postoperative 7.2 ± 2.5°, *p* = n.s.). On the other hand, placement of the opening wedge at any other site resulted in significant changes at both the M and AM levels (postoperative M 8.7 ± 2.8°, *p* = 0.02 and postoperative AM 11.2 ± 3.3°, *p* < 0.0001). The remaining results from the global analysis and the relative differences are presented in Table 2 and Fig. 5.

**Table 2:** Comparison of preoperative and global (3 & 4 cm, n = 12) postoperative PTS

	Mean PTS, SD	***P*** value	Relative difference
**Pre**	7 ± 2.3°		
**Post PM**	7.2 ± 2.5°	Pre vs. post PM 0.7	2.9%
**Post M**	8.7 ± 2.8°	Pre vs. post M 0.02	24%
**Post AM**	11.2 ± 3.3°	Pre vs. post AM < 0.0001	60%

### PTS in the 3 cm group

**Fig. 6:**
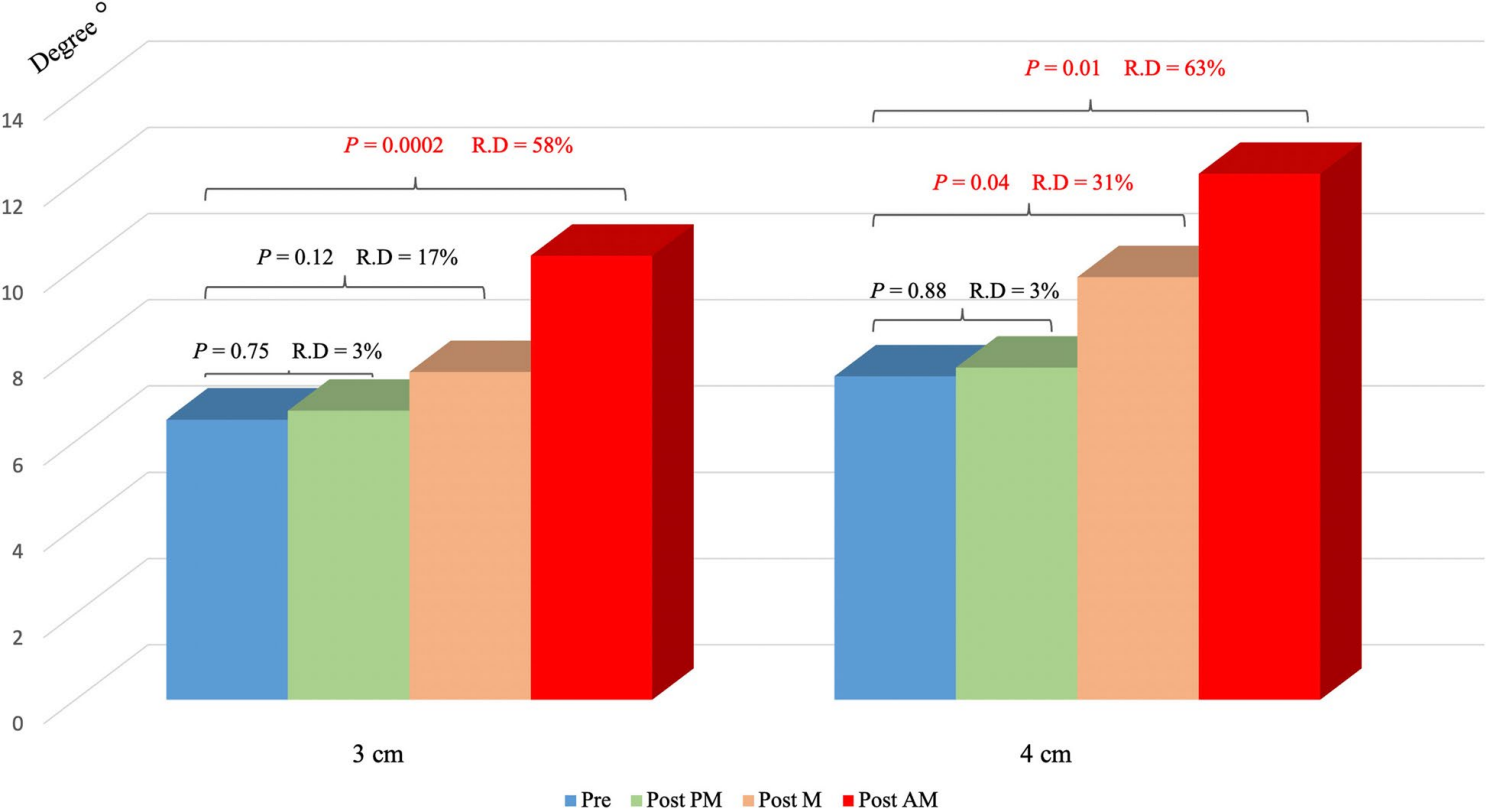
Comparisons of preoperative and postoperative PTS, in the 3 and 4 cm groups. The red text indicates significant changes. R. D, relative difference; AM, anteromedial; M, medial; PM, posteromedial

Based on the analysis of the *3 cm group,* placing the opening wedge in the PM (*p* = n.s.) or M (*p* = n.s.) zone did not significantly alter the resulting PTS, and a relative difference from the preoperative values of 3% and 17%, respectively, was observed (Table 3 and Fig. 6).

**Table 3:** Independent comparisons of preoperative and postoperative PTS in the 3 and 4 cm groups

		Mean PTS, SD	P value	Relative difference
**3 cm**	Pre	6.5 ± 1.9°		
	Post PM	6.7 ± 2.2°	Pre vs. post PM 0.75	3%
	Post M	7.6 ± 2.5°	Pre vs. post M 0.12	17%
	Post AM	10.3 ± 2.5°	Pre vs. post AM 0.0002	58%
**4 cm**	Pre	7.5 ± 2.8°		
	Post PM	7.7 ± 2.9°	Pre vs. post PM 0.88	3%
	Post M	9.8 ± 2.9°	Pre vs. post M 0.04	31%
	Post AM	12.2 ± 4°	Pre vs. post AM 0.01	63%

SD, standard deviation

### PTS in the 4 cm group

The preoperative PTS was only maintained when placing the opening wedge at the PM level (*p* = n.s.). Placement at the M (*p* = 0.04) and AM (*p* = 0.012) levels generated significant changes in the postoperative PTS. The relative differences from the preoperative value were 31 and 63%, respectively, as shown in Table 3 and Fig. 6.

### Mediolateral measurement

The measured mediolateral size of the tibial epiphysis was not correlated with the magnitude of the postoperative modifications of the PTS.

### Measurement reliability

The interrater reliability of the pre- and postosteotomy PTS measurements was “excellent” and is shown in Table 4. The intrarater reliability was also considered “excellent”, with ICC values ≥0.91 for all measurements performed by both evaluators.

**Table 4:** Interrater reliability

	Obs 1	Obs 2	ICC
**PTS Pre**	6.9 ± 2.5°	7.2 ± 2.5°	0.82 (95% CI 0.25–0.90)
**PTS Post PM**	6.8 ± 2.1°	7.6 ± 2.9°	0.92 (95% CI 0.73–0.98)
**PTS Post M**	8.4 ± 3°	9.0 ± 2.8°	0.94 (95% CI 0.81–0.98)
**PTS Post AM**	10.9 ± 3.6°	11.6 ± 3.1°	0.95 (95% CI 0.84–0.99)
**ML**	86.6 ± 3.5	90.1 ± 3.1	0.89 (95% CI 0.77–0.91)

PTS, posterior tibial slope; Pre, preoperative; Post, postoperative; PM, posteromedial; M, medial, AM, anteromedial; ML, mediolateral; Obs, observer; ICC, intraclass correlation coefficient

## Discussion

The most relevant findings of this study reflect the impact on the PTS of distalizing the point where the osteotomy begins, since a greater distance between the beginning of the osteotomy and the joint line results in a greater risk of causing changes in the PTS. Another finding in this study is the importance of placing the opening wedge in the PM third of the tibia when performing an HTO, since significant changes in PTS were not detected in either group (3 or 4 cm) after the procedure. The results of the present study confirm the initial hypothesis.

As previously reported, the PTS tends to increase when performing OWHTO [5]. This increase in the inclination in the sagittal plane is transformed into a greater force of tibial displacement towards the anterior direction, increasing the demand on the native ACL [23]. Likewise, evidence also supports the impact of the PTS on the ACL postreconstruction [24, 25].

A greater lateral PTS in patients who suffered early failure after ACL reconstruction versus those who had satisfactory results after the same reconstruction was shown by Christensen et al. [26, 27].

The role of the medial PTS as a predictive factor for ACL reconstruction failure is more controversial [28, 29]. However, different studies [30–32] are consistent with those published by Webb et al. [10]; in their study, a medial PTS > 12° was stated to increase the risk of failure after ACL reconstruction.

Furthermore, an iatrogenic increase in the PTS not only affects the native or reconstructed ACL but also, as Rodner et al. have shown, generates greater pressure in the posteromedial compartment [33].

According to the findings from the present study, the undesired effects on the ACL and the increased pressure on the posteromedial compartment observed after the increase in the PTS might be avoided by considering certain technical aspects during the performance of an OWHTO, such as the distance from the joint space where the osteotomy begins and the placement of the opening wedge in the PM third of the tibia.

Consistent with the data published by Rubino et al., performing the opening in the PM third of the tibia successfully avoids modifications in the PTS. The findings of our study show that as the opening wedge is moved to the M and AM thirds, the increases in the PTS are progressive (Table 3), similar to the study by Rubino et al. In their study, the placement of the wedge at the level of the M third also showed significant changes in the TS, although the authors did not specify the distance from the joint line where the osteotomy began [34]. According to the results obtained in our study, the height where the osteotomy begins plays a relevant role in the resulting PTS, since no significant differences in the PTS were observed when the opening was created in the medial third in the *3 cm* group (preoperative PTS of 6.5 ± 1.9° vs. postoperative PTS of 7.6 ± 2.5°, relative difference 17%, *p* = n.s.). However, a significant increase in the PTS was observed when the osteotomy began 4 cm from the joint space and the opening was established at the level of the M third (preoperative PTS of 7.5 ± 2.8° vs. postoperative PTS of 9.8 ± 2.9°, relative difference 31%, *p* = 0.04).

The findings presented in our study may be related to the three-triangle method published by Noyes et al. in 2005 [35]. In that study, the medial cortical tibia was established to have an angulation of 45° in the axial plane, and when creating the opening, the anterior gap was suggested to be half of the posterior gap. According to their published data, assuming a medial cortex of the tibia measuring 40 mm, a 10° increase in the PTS would be generated by an error of 5 mm in the size of the anterior gap. It was concluded by the authors that as a general rule, for a 40-mm medial cortex, each millimetre alteration in the opening gap results in a 2° increase in the PTS. It was also established that if the size of the medial cortex decreases to 20 mm, then each millimetre of error in the anterior opening gap generates a 4° increase in the PTS. According to the data from this study and the existing differences in the mediolateral distance at 3 and 4 cm in our series (Table 1), when selecting a distal site to begin the osteotomy, a smaller medial cortical bone is assumed, which is associated with a greater risk of producing increases in the PTS after minimal changes in the placement of the opening wedge [36].

Different authors mention the site they chose to begin the osteotomy, but to our knowledge, these different levels have not been compared with each other to date. Matar et al. preferred the metaphyseal-diaphyseal junction area, distal to the capsular insertion and 3 cm from the joint space, considering this site as the safest since they managed to place a retractor and protect the posterior neurovascular structures [37]. In contrast, Noyes FR et al. prefer starting at 3.5 cm [38], and other authors have started at 4 cm from the joint space [39].

The findings from the present study have an even more relevant implication for those cases where simultaneous ACL reconstruction associated with OWHTO is proposed. In these cases, it was suggested by Cantivalli et al. that OWHTO should begin 4 cm from the joint space to create a tibial tunnel of sufficient length and prevent it from starting within the osteotomy gap [39]. The lower starting point suggested by these authors could generate a clear undesired effect on the PTS if the surgeon does not maintain strict placement of the opening wedge at the PM level. In this context, the opening at the level of the M third would generate an increase in the PTS > 2°, with all the consequences that this change would impose on the recent reconstruction of the ACL [10, 11].

Recently, in another cadaveric study [19], the effect on the PTS of an OWHTO in which the lateral hinge was located 1 cm distal to the standard position was analysed [1, 33, 40]. The authors of this study concluded that the distalization of the lateral hinge generated an increase in the PTS compared to the site defined as a standard to locate it. This distalization of the hinge not only affected the PTS but also generated a greater number of fractures in the lateral cortex of the tibia. These aforementioned changes were attributed by the authors to the decrease in the axial size of the tibia at that level. In our study, cases of fracture were not observed when the standard height of the lateral hinge was chosen. However, when the osteotomy was started 4 cm from the joint space, which represents a smaller ML distance, changes in the PTS occurred more easily, similar to the results published by Jo HS et al. [19].

This study is not without limitations. Because it was a cadaveric study, the number of subjects was limited. However, the number of specimens used is consistent with other cadaver studies focused on the analysis of the tibial slope, whose range is 8–16 knees [1, 13, 19]. Due to the characteristic bone quality of this sample, we did not propose the creation of openings of different amplitudes. These limitations were minimized by selecting the degree of opening based on the protocols used in other studies that analyse the PTS when performing an OWHTO [13]. In addition, because the specimens did not include the ankle joint, the PTS was measured on a lateral radiograph of the knee according to the proximal anatomic axis of the tibia [41]. However, with this methodology, no significant differences in the PTS should be observed if it is measured according to the complete anatomical axis of the tibia on full-length lateral radiographs [42]. Similarly, varus/valgus alignment could not be evaluated due to the lack of a complete limb and the impossibility of performing radiographs in the standing position [43].

Finally, and when also presenting the amputation in the proximal third of the femur, the possible relationship between the different changes produced in the PTS and the patellar height could not be studied due to the interruption of the extensor apparatus.

In terms of the clinical relevance of this work, both the site where the opening is performed and the corresponding wedge is placed and the distance from the joint line where the osteotomy will begin must be considered by the orthopaedic surgeon prior to surgery. In cases requiring an OWHTO associated with ACL reconstruction, the distalization of the site where the osteotomy begins potentially exerts a relevant effect on the PTS.

## Conclusion

Correct control of the posterior tibial slope can be achieved by avoiding a low point when beginning the osteotomy and placing the opening wedge in the posteromedial third of the tibia when performing an opening-wedge high tibial osteotomy.

## Data Availability

The datasets used and/or analysed during the current study are available from the corresponding author on reasonable request.

## Abbreviations

HTO: high tibial osteotomy
OWHTO: opening-wedge high tibial osteotomy
PTS: posterior tibial slope
PH: patellar height
ACL: anterior cruciate ligament
cm: centimetres
AP: anteroposterior
AM: anteromedial
M: medial
PM: posteromedial
ICC: intraclass correlation coefficient
MRI: magnetic resonance imaging.
